# Privacy-Preserving ECC-Based AKA for Resource-Constrained IoT Sensor Networks with Forgotten Password Reset

**DOI:** 10.3390/e28020185

**Published:** 2026-02-06

**Authors:** Yicheng Yu, Kai Wei, Kun Qi, Wangyu Wu

**Affiliations:** 1 School of Electronic and Communication Engineering, Shenzhen Polytechnic University, Shenzhen 518055, China; 2 School of Computer Science, University of Liverpool, Liverpool L69 3DR, UK

**Keywords:** wireless sensor networks, authentication and key agreement, elliptic curve cryptography, physically unclonable function, forgotten password reset

## Abstract

Wireless sensor networks (WSNs) are extensively used in IoT applications. Secure access control and data protection are essential. Nonetheless, the wireless environment has an open nature. The limited resources of sensor devices render WSNs susceptible to a variety of security attacks, causing significant difficulties in the design phase of efficient authentication and key agreement (AKA) protocols. This study proposes a physically unclonable function (PUF)-based lightweight and secure AKA protocol for WSNs based on elliptic curve cryptography (ECC). A secure password update scheme is offered, which would allow legitimate users to reset forgotten passwords without re-registration. According to formal security analysis using BAN logic and ProVerif, the proposed protocol is secure against common attacks. Moreover, from an entropy perspective, the use of dynamic pseudonyms and fresh session randomness increase an adversary’s uncertainty about user identities, thereby limiting identity-related information leakage. Performance evaluation shows that the proposed protocol achieves lower computational and communication overhead than the existing ones, making it suitable for WSNs with resource constraints.

## 1. Introduction

WSNs are used in many scenarios, for example, monitoring the environment, enabling healthcare-related services, supporting automated industrial processes, and improving transportation intelligence [1,2]. In this case, a large number of sensor nodes are used for collecting sensitive data and sending those data to authentic users using wireless communication channels. The open nature of wireless transmission, the unattended deployment at the sensor node’s end, and the limited physical resources or capabilities of the sensor nodes make WSN a prime target of various security issues like spoofing of node identities, replay-based intrusion, communication hijacking, and offline guessing of authentication secrets, etc. [3,4,5,6].

Authentication and Key Agreement (AKA) protocols are therefore indispensable components for securing WSN communications. A well-designed AKA protocol which guarantees that only legitimate users and sensor nodes are able to access the network service and fresh session keys are established to secure the transmission of further packets. Nonetheless, the task of designing efficient and secure AKA protocols for WSNs is challenging. On the one hand, sensor nodes have limited computation capability, memory, and battery power, which restricts the adoption of heavyweight cryptographic techniques. On the other hand, AKA protocols are required to provide strong security properties, which not only offer mutual authentication but also privacy preservation, perfect forward secrecy, and robustness against known attacks under powerful adversaries [7,8]. In particular, privacy in IoT-based authentication can be intuitively understood from an entropy perspective, where higher uncertainty of user identities given the observed protocol messages implies stronger resistance to tracing and identity leakage.

In recent years, several AKA schemes have been suggested to tackle these issues. Due to their ability to offer high security levels while using relatively small key sizes, elliptic curve cryptography (ECC) has become increasingly popular in recent years. This makes ECC suitable for WSNs, which often operate under severe resource constraints [3]. Moreover, hardware security mechanisms which use techniques such as PUFs (Physically Unclonable Functions) have been deployed to protect sensor node secrets from hardware cloning attacks [8]. The development of quantum computing has led to the emergence of post-quantum secure AKA protocols which can potentially offer long-term security of WSNs against a quantum gains adversary [4].

**Figure 1:**
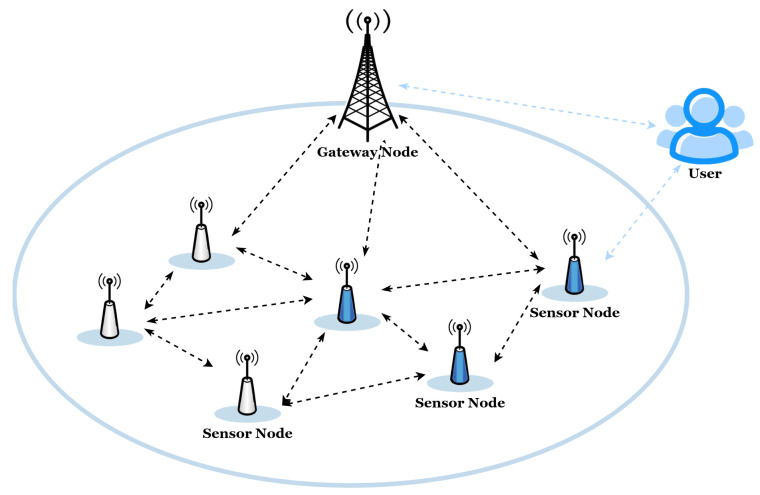
A typical WSN scenario.

Figure 1 illustrates a typical WSN application. Three main components are involved: users, gateway nodes, and sensor nodes. Within such a framework, the sensed data are accessed by the users through gateway nodes that connect the users with the sensor nodes. Sensor nodes cooperatively perform data sensing and forwarding tasks, while the gateway node assists in authentication, access control, and key establishment. A practical WSN deployment basically follows this interaction pattern that also shows the importance of using secure and lightweight AKA protocols to protect the communications between various entities.

In light of these observations, we present a secure and efficient AKA protocol for WSNs using ECC-based cryptographic techniques, smart-card-assisted user authentication, and PUF-based security of sensor nodes. We aim for strong security properties alongside lightweight computation and modest communication overhead. By combining BAN logic analysis, ProVerif security verification, and efficiency evaluation, we confirm that the proposed scheme can be applied effectively in practical WSN environments.

The major contributions are as follows:

- We present a lightweight AKA protocol for WSNs which achieves mutual authentication and establishes a secure session key.
- A PUF-based mechanism is deployed to enhance resistance of sensor nodes against physical attacks.
- The proposed protocol and its security properties are analyzed using BAN logic and ProVerif.
- We evaluate the computational and communication costs and demonstrate the efficiency of the proposed scheme compared with existing related protocols.
- We design a secure and user-friendly password update mechanism that allows legitimate users to reset forgotten passwords without requiring re-registration [9], while ensuring the overall security of the system remains intact.

## 2. Related Works

Due to various constraints such as limited computational power, energy constraints, and open wireless channels, the establishment of a secure session is very challenging. To overcome the challenges, AKA protocols are applied. Many lightweight and robust AKA schemes with strong security characteristics like mutual authentication, anonymity, forward secrecy, low computation, and communication overhead have been proposed over recent years.

A significant piece of work explores the use of Elliptic Curve Cryptography for efficient authentication. Huang et al. developed a three-factor ECC-based AKA protocol which uses biometrics, smart card, and password with formal security verification and attack resistance, which is ideally suited to resource-constrained environments [3]. To reduce overhead and improve anonymity, Li and Hu’s lightweight ECC-based AKA protocol resists ephemeral secret leakage attack [7]. Many ECC-based designs have also been proposed which can withstand offline guessing or replay attack during the session key negotiation [10]. Simultaneously, protocol proposals for lightweight two-factor AKA have occurred that are based on chaotic and symmetric which are provably secure and lightweight [11].

Hybrid approaches that combine symmetric and asymmetric mechanisms to optimize energy consumption and throughput have also been explored. For instance, secure hybrid data transmission protocols integrate key management and message authentication to support efficient node authentication and data integrity [12]. Broader surveys of IoT security and authentication mechanisms highlight the limitations of existing AKA protocols and underscore the ongoing need for lightweight schemes that maintain strong security features in diverse deployment contexts [13].

Architectural innovations such as multi-gateway AKA schemes seek to improve scalability and flexibility in WSNs. Yang et al. present a multi-gateway structure that allows dynamic access for sensors and users across network regions with reduced computation and communication costs relative to earlier schemes [14]. Beyond structural enhancements, hardware-assisted authentication mechanisms using Physically Unclonable Functions (PUFs) have been investigated. Tyagi and Kumar introduce PUF-based AKA protocols combined with ECC to bolster resistance against smart card loss and physical attacks [8], and further work integrates PUF with chaotic maps to achieve dynamic pseudonym generation and resistance to modeling attacks [15].

The emergence of post-quantum secure AKA schemes is due to quantum computing threats. Singh and Mishra suggest an AKA protocol for resisting a quantum attacker that relies on the security of Ring Learning With Errors (RLWE) [4]. Advanced frameworks combine lightweight cryptography with context-aware key management to adapt AKA to both classical and post-quantum threat environments [16].

Aside from these particular designs, smart-card based authentication and fuzzy-extractor-based authentication schemes defend against card loss and impersonation attacks with minimal overhead to sensor nodes [17]. Together, these works demonstrate the recent diversity of WSN AKA research on ECC-based multi-factor schemes, lightweight symmetric and chaotic protocols, multi-gateway structures, PUF-assisted schemes, and post-quantum view.

## 3. Preliminaries

### 3.1. System Model

**Figure 2:**
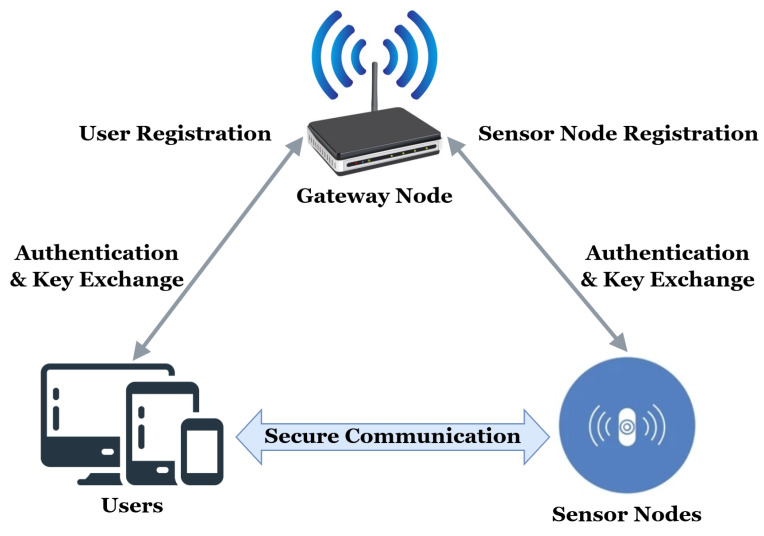
System model.

The system model of the proposed AKA protocol is illustrated in Figure 2. The wireless sensor network consists of three types of entities: users $U_{i}$, a gateway node GW, and multiple sensor nodes $S_{j}$.

The trusted authority is the gateway node responsible for initializing the network, registering users, and registering sensor nodes. In the registration stage, the user as well as sensor nodes submit their identity-related information to the gateway in a secure manner which provides and distributes the corresponding credentials. Once deployed, the gateway facilitates the authentication and key agreement between the users and sensor nodes but not in data transmission.

A user can access the sensed data via the gateway after performing mutual authentication and session key establishment of target sensor nodes. On successful authentication, a session key is established between the user and the sensor node for designated secure communication, as shown in Figure 2.

### 3.2. Threat Model

The protocol’s security analysis follows the Dolev–Yao adversarial model [18]. The adversary is assumed to have complete control of the public communication channel, where they can eavesdrop, intercept, modify, replay, and forge messages. However, the adversary cannot break standard cryptographic primitives, such as one-way hash functions and elliptic curve cryptographic operations.

Moreover, the adversary can physically take over the sensor nodes or steal user smart cards to get the stored data. Nonetheless, it is assumed that it is infeasible for malicious parties to extract sensitive secrets from cryptographic primitives, PUFs or solve the elliptic curve discrete logarithm problem.

### 3.3. Cryptographic Primitives

- **One-way hash function**: We adopt a cryptographic h(·) to derive authentication values and maintain message integrity. It is assumed to be collision-resistant and pre-image-resistant.
- **Elliptic Curve Cryptography (ECC)**: Consider an elliptic curve $E(F_{p})$ over the finite field $F_{p}$. Let *G* denote a cyclic additive group of prime order *q* generated by *P*. The security of the ECC-based computations is grounded on the intractability of the elliptic-curve discrete logarithm problem (ECDLP).
- **Physical Unclonable Function (PUF)**: A PUF [19] is a hardware-rooted primitive that outputs device-unique, hard-to-predict responses for supplied challenges. It is utilized to protect sensor node secrets against physical attacks and cloning.

## 4. Proposed Protocol

The proposed protocol comprises six main phases: the initialization phase, the user registration phase, the sensor node registration phase, the mutual authentication phase, the password update phase, and the forgotten password reset phase. The gateway node executes the operations required in the first three phases. The mutual authentication phase is designed to achieve bidirectional identity verification between the user and the sensor node, and to negotiate a secure session key for ensuring the confidentiality and integrity of subsequent communications. If a password change is needed, the password update phase allows the password to be modified securely. In addition, for scenarios where a user forgets the password, the protocol provides a forgotten password reset phase, allowing the user to reset the password without providing the original one.

### 4.1. Initialization Phase

During the system initialization phase, the gateway node GW performs the offline setup of relevant parameters and selects security functions. GW selects an elliptic curve *E* defined over a finite field Fp, and chooses an additive group *G* on *E* of order *q*, where *P* denotes a generator of *G*. Then, GW generates a private key *x* and computes the corresponding public key X=xP. Finally, GW chooses a master key *K* and a secure cryptographic hash function $h\left(.\right):{\left\{0,1\right\}}^{*}\to {\left\{0,1\right\}}^{256}$. The public parameters E(Fp),G,P,X,h(.) are subsequently published.

### 4.2. User Registration Phase

**Figure 3:**
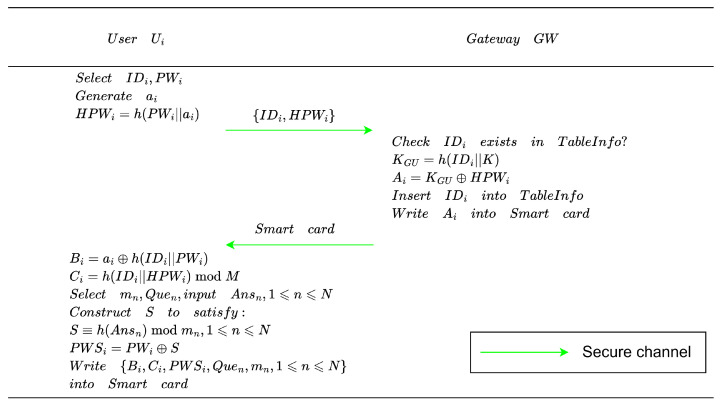
User registration phase.

Before obtaining sensor-collected data, a user is required to enroll with the gateway. The registration steps are outlined below. When a user needs to access data collected by sensor nodes in the system, they must first complete registration with the gateway node. The process of the user registration phase is shown in Figure 3 and outlined below:

- **Credential Setup**. User $U_{i}$ chooses an identity $ID_{i}$ and a corresponding password $PW_{i}$, and generates a random number $a_{i}$. $U_{i}$ then computes $HPW_{i}=h(PW_{i}\left|\right|a_{i})$ and sends the parameter $\left\{ID_{i},HPW_{i}\right\}$ to the gateway GW over a secure channel. After obtaining $\left\{ID_{i},HPW_{i}\right\}$, GW verifies whether $ID_{i}$ is already registered. If it exists, $U_{i}$ is requested to choose a different identity; otherwise, GW computes $K_{GU}=h(ID_{i}\left||K\right)$ and $A_{i}=K_{GU}⊕HPW_{i}$, stores $ID_{i}$ in the user table, writes $A_{i}$ into a smart card SC, and delivers SC securely to $U_{i}$.
- **Local Verification Setup**. After receiving SC, $U_{i}$ computes $B_{i}=a_{i}⊕h(ID_{i}||PW_{i})$ and $C_{i}=h(ID_{i}||HPW_{i})\;\mathrm{mod}\;M$. *M* is chosen as a sufficiently large integer (e.g., $M=2^{k}$, where 64≤k≤128) to prevent efficient offline verification of guessed ($ID_{i}$, $PW_{i}$) pairs.
- **Forgotten Password Reset Support**. To support scenarios where the user forgets the password, the protocol further provides a forgotten password reset initialization based on security questions. User $U_{i}$ selects *N* pairwise coprime positive integers $m_{1}$, $m_{2}$, …, $m_{N}$, and *N* security questions $Que_{1}$, $Que_{2}$, …, $Que_{N}$, then provides the corresponding answers $Ans_{1}$, $Ans_{2}$, …, $Ans_{N}$. Subsequently, a secret *S* is constructed based on the Chinese Remainder Theorem such that it satisfies: $S\equiv h\left(Ans_{n}\right)\;\mathrm{mod}\;m_{n},1⩽n⩽N$. Finally, $U_{i}$ computes $PWS_{i}=PW_{i}⊕S$ and writes the parameters $\left\{B_{i},C_{i},PWS_{i},Que_{n},m_{n},1⩽n⩽N\right\}$ into SC.

### 4.3. Sensor Node Registration Phase

**Figure 4:**
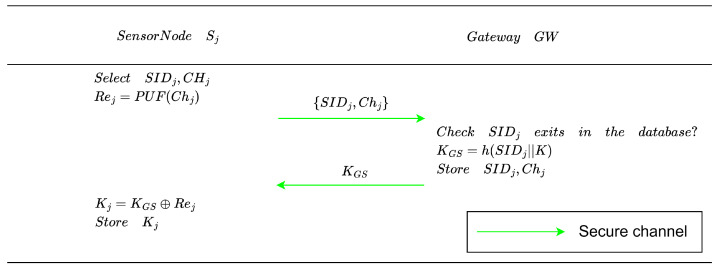
Sensor node registration phase.

Prior to deployment in the operational area, a new sensor node $S_{j}$ must also complete registration with GW. The sensor registration phase binds each sensor node to the gateway and establishes a long-term credential protected by the PUF, which prevents physical cloning attacks. The sensor node registration process is shown in Figure 4, and the specific steps are described below:

- **Registration request**. The sensor node $S_{j}$ picks an identity $SID_{j}$ and a random challenge $Ch_{j}$, then generates the corresponding response $Re_{j}$ via a Physical Unclonable Function (PUF). Subsequently, $S_{j}$ transmits $SID_{j}$ and $Ch_{j}$ to GW over a secure channel.
- **Gateway credential assignment**. Upon receiving $SID_{j}$ and $Ch_{j}$, GW first verifies whether the same $SID_{j}$ is already present in the sensor node information table. If a duplicate is found, $SID_{j}$ is notified to regenerate its identity information. Otherwise, GW computes the key $K_{GS}=h(SID_{j}\left||K\right)$, records $SID_{j}$ and $Ch_{j}$ in the sensor node information table, and finally sends $K_{GS}$ back to $S_{j}$ through the secure channel. With $K_{GS}$, $S_{j}$ computes its local key $K_{j}$ and stores it securely.

### 4.4. Mutual Authentication Phase

When a user needs to access data collected by sensor nodes, both the user and the sensor node must perform bidirectional authentication with the assistance of the gateway node. A shared session key is also negotiated to ensure the confidentiality of subsequent communications. Since the long-term credentials are established during registration, mutual authentication is completed through four message exchanges ($Msg_{1}$–$Msg_{4}$), as follows:

- **${\mathrm{Msg}}_{1}$: User ⇒ Gateway (Login request)**. The user $U_{i}$ inputs the identity $ID_{i}$ and corresponding password $PW_{i}$, and inserts the smart card into the terminal. The terminal first performs local password verification by computing:
  $$a_{i}^{*}=B_{i}⊕h(ID_{i}||PW_{i}),\;HPW_{i}^{*}=h(PW_{i}\left|\right|a_{i}^{*}),\;C_{i}^{*}=h(ID_{i}||HPW_{i}^{*})\;\mathrm{mod}\;M,$$
  and verifies whether $C_{i}^{*}$ matches $C_{i}$. If they do not match, the terminal rejects the login request. Otherwise, the terminal obtains the current timestamp $T_{1}$, generates random numbers *w* and $r_{i}$, selects the identity $SID_{j}$ of the sensor node to be accessed. It successively computes:
  $$K_{GU}=A_{i}⊕HPW_{i},\;D_{i}=w\cdot P,\;E_{i}=w\cdot X,\;R_{i}=r_{i}\cdot P,\;PID_{i}=ID_{i}⊕E_{i},\;M_{1}=(R_{i}||SID_{j})⊕K_{GU},\;M_{UG}=h(ID_{i}\left|\right|R_{i}\left|\right|K_{GU}\left|\right|M_{1}\left|\right|T_{1}).$$
  Finally, the terminal sends:
  $$Msg_{1}=\left\{PID_{i},D_{i},M_{1},M_{UG},T_{1}\right\}$$
  to the gateway node GW.
- **${\mathrm{Msg}}_{2}$: Gateway ⇒ Sensor (Authentication challenge)**. Upon receiving $Msg_{1}$, GW first verifies the validity of timestamp $T_{1}$, then uses its private key *x* to compute:
  $$E_{i}^{*}=x\cdot D_{i},\;ID_{i}^{*}=PID_{i}⊕E_{i}^{*}.$$
  GW checks the validity of $ID_{i}^{*}$ by querying the user information table, then computes:
  $$K_{GU}=h(ID_{i}^{*}\left||K\right),\;(R_{i}^{*}||SID_{j})=M_{1}⊕K_{GU},\;M_{UG}^{*}=h(ID_{i}^{*}\left|\right|R_{i}^{*}\left|\right|K_{GU}\left|\right|M_{1}\left|\right|T_{1}).$$
  GW compares whether $M_{UG}^{*}$ equals $M_{UG}$. If they are not equal, GW terminates the session. Otherwise, GW generates timestamp $T_{2}$, set $ID_{i}=ID_{i}^{*}$, $R_{i}=R_{i}^{*}$, and computes:
  $$K_{GS}=h(SID_{j}\left||K\right),\;M_{2}=(ID_{i}\left|\right|R_{i})⊕K_{GS},\;M_{GS}=h(ID_{i}||SID_{j}\left|\right|R_{i}\left|\right|K_{GS}\left|\right|T_{2}).$$
  GW retrieves the challenge $Ch_{j}$ and sends:
  $$Msg_{2}=\left\{M_{2},M_{GS},Ch_{j},T_{2}\right\}$$
  to $S_{j}$.
- **${\mathrm{Msg}}_{3}$: Sensor ⇒ Gateway (Sensor response)**. After receiving $Msg_{2}$, sensor node $S_{j}$ first checks the validity of timestamp $T_{2}$. Then, it generates its PUF response and computes:
  $$K_{GS}=K_{j}⊕PUF\left(Ch_{j}\right),\;(ID_{i}\left|\right|R_{i})=M_{2}⊕K_{GS},\;M_{GS}^{*}=h(ID_{i}||SID_{j}\left|\right|R_{i}\left|\right|K_{GS}\left|\right|T_{2}).$$
  If $M_{GS}^{*}\neq M_{GS}$ the authentication session is terminated. Otherwise, $S_{j}$ generates random number $r_{j}$ and computes:
  $$R_{j}=r_{j}\cdot P,\;SK_{ji}=h(R_{i}\left|\right|R_{j}\left|\right|r_{j}\cdot R_{i}),\;M_{3}=(R_{j}||ID_{i}||SID_{j})⊕K_{GS},\;M_{SG}=h(SID_{j}||ID_{i}\left|\right|R_{j}\left|\right|T_{3})$$
  where $T_{3}$ is the current timestamp and $SK_{ji}$ is the session key. Finally, $S_{j}$ returns
  $$Msg_{3}=\left\{M_{3},M_{SG},T_{3}\right\}$$
  to GW.
- **${\mathrm{Msg}}_{4}$: Gateway ⇒ User (Session confirmation)**. Upon receiving $Msg_{3}$, GW checks the validity of timestamp $T_{3}$ and computes:
  $$(R_{j}||ID_{i}||SID_{j})=M_{3}⊕K_{GS},\;M_{SG}^{*}=h(SID_{j}||ID_{i}\left|\right|R_{j}\left|\right|T_{3}).$$
  If $M_{SG}^{*}$ matches $M_{SG}$, GW obtains the current timestamp $T_{4}$ and computes:
  $$M_{4}=(R_{j}||SID_{j})⊕K_{GU},\;M_{GU}=h(ID_{i}\left|\right|R_{j}\left|\right|K_{GU}\left|\right|T_{4}),$$
  and returns the message:
  $$Msg_{4}=\left\{M_{4},M_{GU},T_{4}\right\}$$
  to user $U_{i}$. After receiving $Msg_{4}$, $U_{i}$ checks the validity of timestamp $T_{4}$, and computes:
  $$(R_{j}||SID_{j})=M_{4}⊕K_{GU},\;M_{GU}^{*}=h(ID_{i}\left|\right|R_{j}\left|\right|K_{GU}\left|\right|T_{4}).$$
  If $M_{GU}^{*}$ is verified to match $M_{GU}$, $U_{i}$ generates the session key:
  $$SK_{ij}=h(R_{i}\left|\right|R_{j}\left|\right|r_{i}\cdot R_{j}),$$
  thereby completing mutual authentication with sensor node $S_{j}$.

**Figure 5:**
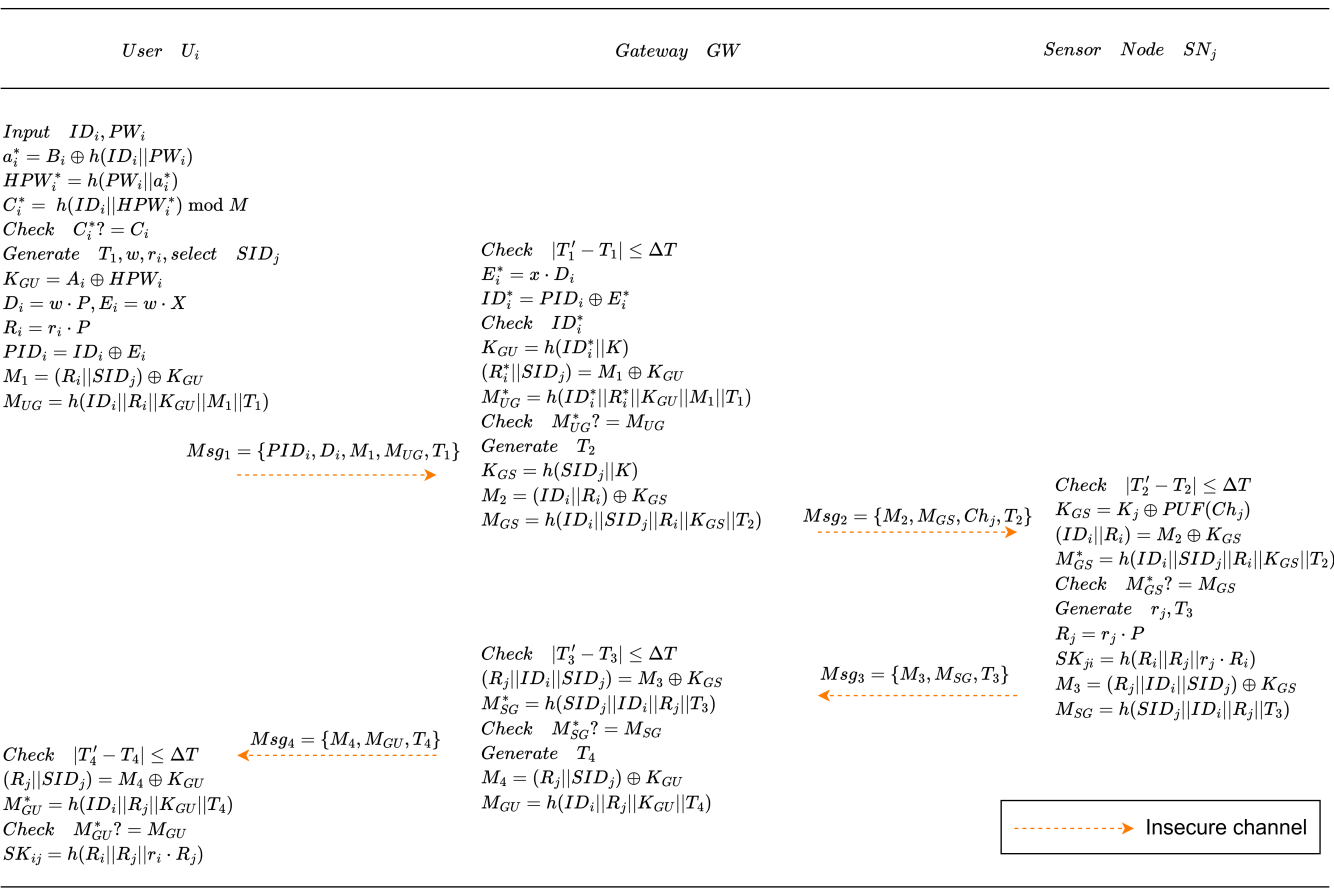
Mutual authentication phase.

The process of the mutual authentication phase described above is illustrated in Figure 5.

### 4.5. Password Update Phase

**Figure 6:**
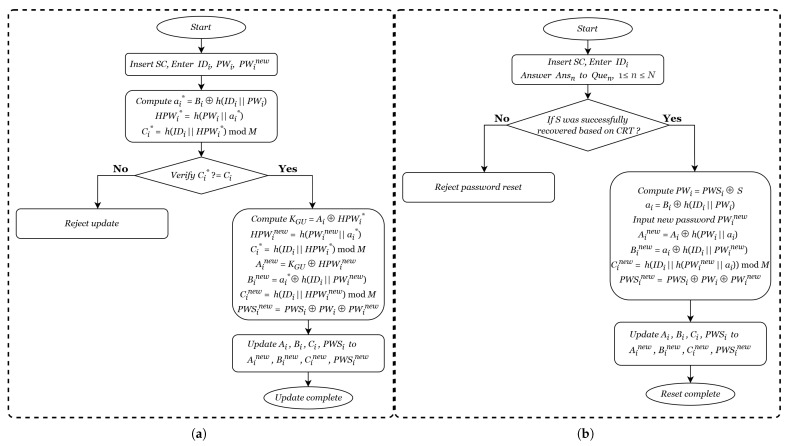
(**a**) Password update phase. (**b**) Forgotten password reset phase.

During the password update phase, the user can modify their password offline without interacting with the gateway node. Figure 6 is a flowchart of the process, and the specific steps are as follows:

- User $U_{i}$ inserts SC into a terminal and enters the identity $ID_{i}$, the original password $PW_{i}$, and the new password $PW_{i}^{new}$.
- The terminal computes $a_{i}^{*}=B_{i}⊕h(ID_{i}||PW_{i})$, $HPW_{i}^{*}=h(PW_{i}\left|\right|a_{i}^{*})$, and $C_{i}^{*}=h(ID_{i}||HPW_{i}^{*})\;\mathrm{mod}\;M$, and verifies whether the stored $C_{i}$ in the smart card matches the computed $C_{i}^{*}$. If they do not match, the password update request is rejected. Otherwise, the terminal successively computes $K_{GU}=A_{i}⊕HPW_{i}^{*}$, $HPW_{i}^{new}=h(PW_{i}^{new}\left|\right|a_{i}^{*})$, $A_{i}^{new}=K_{GU}⊕HPW_{i}^{new}$, $B_{i}^{new}=a_{i}^{*}⊕h(ID_{i}||PW_{i}^{new})$, $C_{i}^{new}=h(ID_{i}||HPW_{i}^{new})\;\mathrm{mod}\;M$, and $PWS_{i}^{new}=PWS_{i}⊕PW_{i}⊕PW_{i}^{new}$, and updates the original parameters $A_{i}$, $B_{i}$, $C_{i}$, and $PWS_{i}$ in the smart card to $A_{i}^{new}$, $B_{i}^{new}$, $C_{i}^{new}$ and $PWS_{i}^{new}$, respectively.

### 4.6. Forgotten Password Reset Phase

When a user forgets their password and needs to log in, they can securely restore access through the following procedure, as shown in Figure 6.

- The user $U_{i}$ inserts SC into a terminal, enters the correct identity $ID_{i}$, and provides accurate answers $Ans_{n}$ to all preset security questions $Que_{n}$, 1⩽n⩽N, thereby recovering the secret value *S* based on the Chinese Remainder Theorem (CRT).
- Subsequently, $U_{i}$ computes the original password $PW_{i}=PWS_{i}⊕S$ and the parameter $a_{i}=B_{i}⊕h(ID_{i}||PW_{i})$, inputs the new password $PW_{i}^{new}$, and successively calculates $A_{i}^{new}=A_{i}⊕h(PW_{i}\left|\right|a_{i})⊕h(PW_{i}^{new}\left|\right|a_{i})$, $B_{i}^{new}=a_{i}⊕h(ID_{i}||PW_{i}^{new})$, $C_{i}^{new}=h(ID_{i}||h(PW_{i}^{new}\left|\right|a_{i}))\;\mathrm{mod}\;M$, and $PWS_{i}^{new}=PWS_{i}⊕PW_{i}⊕PW_{i}^{new}$. Finally, the stored values $A_{i}$, $B_{i}$, $C_{i}$ and $PWS_{i}$ in SC are updated to the newly computed $A_{i}^{new}$, $B_{i}^{new}$, $C_{i}^{new}$, and $PWS_{i}^{new}$, respectively, thereby completing the secure reset of the forgotten password to $PW_{i}^{new}$.

## 5. Security Analysis

### 5.1. Formal Security Analysis

We evaluate the proposed AKA protocol through two widely used formal methods: BAN logic and ProVerif. Using BAN logic, we prove that mutual authentication holds and that a session key is successfully set up. Essential security requirements (e.g., session-key confidentiality and authentication) are then machine-checked in ProVerif under the Dolev–Yao adversary model. The two complementary approaches provide a formal assessment of the protocol’s security that is comprehensive and rigorous.

#### 5.1.1. BAN Logic-Based Security Analysis

BAN logic is a belief-based reasoning framework that is commonly adopted to analyze authentication protocols [20]. A set of inference rules abstract actual protocol messages into their idealized form and derive the principals’ beliefs. For convenience, the notation set and the main inference rules are listed in Table 1 and Table 2 separately. Likewise, the idealized messages, assumptions, and step-by-step derivations allowing the authentication goal to be realized are summarized in Table 3. This reasoning shows that $U_{i}$ and $S_{j}$ each accept a newly generated session key and also accept that the other party accepts it, which implies mutual authentication and a secure key-agreement outcome.

#### 5.1.2. ProVerif-Based Formal Verification

To strengthen the security claims, we employ ProVerif [21] to perform automated formal verification under the Dolev–Yao adversary model, where the adversary can fully manipulate the public channel. The ProVerif code of the protocol is publicly available at [22].

In the ProVerif model, the user, gateway, and sensor node are specified as concurrent processes communicating over a public channel. Fresh nonces and session-related parameters are generated using *new*, while cryptographic operations are abstracted as symbolic functions. To model authentication behavior, event statements are inserted at key protocol stages, where *UGbegin(t)*, *UGend(t)*, *GUbegin(t)*, and *GUend(t)* denote the initiation and completion of the user–gateway authentication, and *GSbegin(t)*, *GSend(t)*, *SGbegin(t)*, and *SGend(t)* represent the corresponding gateway–sensor interactions. These events enable ProVerif to reason about agreement between protocol participants.

Authentication is verified using injective correspondence queries, such as *query inj-event(UGend(t)) ==> inj-event(UGbegin(t))*, which assert that whenever a party completes a session, there exists a unique matching session initiation by the peer, thereby providing strong resistance against replay and impersonation attacks. In addition, secrecy properties are specified using confidentiality queries of the form *query not attacker(x)*.

**Figure 7:**
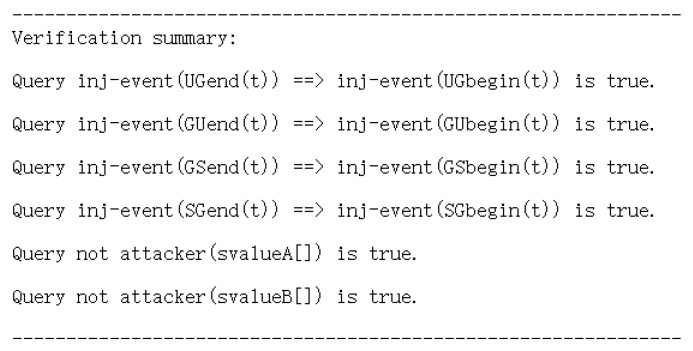
Formal verification results of the proposed protocol using ProVerif.

As shown in Figure 7, all authentication and secrecy queries are successfully verified by ProVerif. Specifically, the results confirm injective authentication between the user, gateway, and sensor node in all protocol phases, as each end event is associated with a unique corresponding begin event. Moreover, the secrecy queries *not attacker(svalueA[])* and *not attacker(svalueB[])* are satisfied, indicating that the attacker cannot derive the modeled sensitive values from protocol executions. Therefore, the ProVerif verification results in Figure 7 provide formal evidence that the scheme achieves mutual authentication and preserves the confidentiality of critical security parameters under an active adversary model.

### 5.2. Informal Security Analysis

In this part, we present an informal security discussion for the proposed authentication scheme. The analysis demonstrates that the protocol achieves the desired security goals and withstands various well-known attacks under the Dolev–Yao adversarial model, where an adversary can observe, intercept, alter, replay, or forge public-channel messages, but is assumed unable to compromise standard cryptographic primitives.

#### 5.2.1. User Anonymity

The actual identity $ID_{i}$ of the user $U_{i}$ is never transmitted in plaintext on the public channel. Instead, we set a dynamic pseudonym $PID_{i}=ID_{i}⊕E_{i}$, where $E_{i}=w\cdot X$ is a random number (dependent on the session) with the public key (system) *X*. Since *w* is freshly chosen in each authentication session, the value of $PID_{i}$ changes dynamically even for the same user. An external adversary cannot derive $E_{i}$ or decipher the real identity $ID_{i}$ without knowledge of the private key *x* of GW. Thus, the proposed protocol preserves user anonymity against both passive eavesdroppers and active adversaries.

#### 5.2.2. Untraceability

The protocol achieves untraceability by making sure that all user-related authentication parameters are not the same for different sessions. In particular, the messages that are transmitted include session-specific random elliptic curve points $R_{i}=r_{i}\cdot P$ and dynamic pseudonyms $PID_{i}$ using fresh random values $r_{i}$ and *w*. Moreover, authentication messages such as $M_{UG}=h(ID_{i}\left|\right|R_{i}\left|\right|K_{GU}\left|\right|M_{1}\left|\right|T_{1})$ bind the identity-related information with timestamps and ephemeral randomness. Since these parameters are statistically independent across sessions, an adversary cannot correlate multiple executions of the protocol to trace a specific user even if all communication messages are recorded.

#### 5.2.3. Mutual Authentication

Through the chained checking of authentication messages, protected by system master key derivatives, the protocol enables mutual authentication among the user, the gateway, and the sensor node. The gateway authenticates the user by validating $M_{UG}$, where $K_{GU}=h(ID_{i}\left||K\right)$ and *K* is the gateway’s master key. Only the holder of *K* must have an ability to generate a valid $M_{UG}$. The sensor node validates the gateway by evaluating $M_{GS}=h(ID_{i}||SID_{j}\left|\right|R_{i}\left|\right|K_{GS}\left|\right|T_{2})$. Here, $K_{GS}=h(SID_{j}\left||K\right)$, which was securely recovered using the sensor’s secret key and PUF response. Lastly, the user authenticates the gateway by verifying the message $M_{GU}$, which includes the fresh sensor-generated value $R_{j}$. As a result, strong mutual authentication will be established among all entities, which will detect any forged and/or modified authentication message.

#### 5.2.4. Session Key Agreement

Once mutual authentication completes, the user and the sensor node independently compute a shared session key. The user computes $SK_{ij}=h(R_{i}\left|\right|R_{j}\left|\right|r_{i}\cdot R_{j})$, while the sensor computes $SK_{ji}=h(R_{i}\left|\right|R_{j}\left|\right|r_{j}\cdot R_{i})$. Due to the properties of elliptic curve scalar multiplication, $r_{i}\cdot R_{j}=r_{j}\cdot R_{i}$. Since the ephemeral secrets $r_{i}$ and $r_{j}$ are never transmitted over the public channel, an adversary cannot derive the session key even with full access to all exchanged messages.

#### 5.2.5. Perfect Forward Secrecy

The proposed protocol offers perfect forward secrecy: each session key is derived from ephemeral random values $r_{i}$ and $r_{j}$, which never get reused in different protocol executions. Even if long-term secrets of the user, sensor node or gateway (i.e., *K*, *x*, or stored credentials) are compromised at some stage, session keys established prior are secure, as recovering those would necessitate solving the ECDLP to extract ephemeral secrets from the past.

#### 5.2.6. Forgotten Password Reset

A secure forgotten password reset mechanism is supported by the protocol. After successfully reconstructing the secret using the pre-selected security credential, the user can reset the password. Also, a password-related verifier is not revealed over the public channel. The reset process does not recover the old password; it does not destroy long-lived secrets or previous session keys. Consequently, the suggested scheme offers a secure approach for resetting forgotten passwords.

#### 5.2.7. Resistance to Impersonation Attack

An adversary generates a valid authentication value $M_{UG}$ to impersonate a legitimate user. This requires knowing $K_{GU}$, along with a fresh random value. It is impossible if the gateway’s master key is inaccessible. In a similar context, the impersonation of a sensor node needs the generation of valid $M_{GS}$ and $M_{SG}$. These values depend on $K_{GS}$ that can be recovered with the help of a sensor’s PUF and its secret key. In conclusion, the protocol efficiently safeguards against impersonation attempts.

#### 5.2.8. Resistance to Stolen Smart Card Attack

The adversary cannot get any other information from the stolen smart card other than some hashed and masked parameters. Credential verification must pass local verification with the correct password; it is also necessary to execute the protocol successfully to generate valid gateway-authenticated messages. This means that having a smart card by itself may not impersonate the user. Thus, it may resist stolen smart card attacks.

#### 5.2.9. Resistance to Offline Password Guessing Attack

The scheme prevents offline password-guessing attacks. Even when an adversary obtains smart-card–stored data through a physical access attack or a side-channel attack, it is still not feasible to uniquely verify guessed identity–password pairs $\left(ID_{i},PW_{i}\right)$ offline. The local verification value $C_{i}$ is obtained using the modular operator, preventing an adversary from efficiently verifying guessed identity–password pairs offline. As a result, an adversary must try online login attempts to find out if a guess pair is correct. The gateway can easily detect and restrict such online attempts through monitoring and access control mechanisms. As a consequence, the proposed scheme effectively prevents offline password guessing attacks and confines the adversary to detectable online attacks.

#### 5.2.10. Resistance to Known Session-Specific Temporary Information Attack

Even if all session-specific temporary data (including $r_{i}$ and $r_{j}$) are revealed, the impact is limited to that session only. The session key $SK=h(R_{i}||R_{j}||r_{i}\cdot R_{j})$ is created from session-dependent values beside the long-term secrets *K*, *x*, $K_{j}$, which include the gateway master key, gateway private key, and sensor secret, respectively, which remain secured. Because new random values are generated with every session, one session’s compromise does not compromise the security of any other session. As such, the designed protocol withstands attacks that rely on exposure of session-only temporary information.

#### 5.2.11. Resistance to Replay Attack

Every authentication message contains timestamps ($T_{1}$, $T_{2}$, $T_{3}$, $T_{4}$) and fresh random numbers. Before processing received messages, the gateway, sensor, and user validate their freshness. Any replayed messages will fail to satisfy the timestamp verification or the authentication hash check, so replay attacks are prevented.

## 6. Performance Analysis

We assess the protocol’s performance in terms of computational complexity and communication overhead. Since the initialization, registration, and password update phases are executed infrequently, the performance evaluation primarily focuses on the mutual authentication phase, which represents the most critical operation in practical deployments. Furthermore, we compare our protocol with the ECC-based three-factor AKA scheme by Huang et al. [3], the industrial IoT authentication protocol by Zhao et al. [23], the anonymous signature-based scheme of Vangala et al. [24], and the privacy-controlled ECC protocol REPACA proposed by Kumar et al. [25].

### 6.1. Computational Performance Analysis

During the mutual authentication phase, three entities are involved: the user $U_{i}$, the gateway node GW, and the sensor node $S_{j}$. The dominant cryptographic operations performed by these entities include: One-way hash operation, and elliptic curve scalar point multiplication, denoted as $T_{h}$ and $T_{M}$, respectively. Other operations, such as XOR, concatenation, and timestamp comparison, incur negligible computational cost compared to $T_{h}$ and $T_{M}$ and are therefore excluded from the analysis.

During the authentication phase, the user performs the following operations:

- Three elliptic curve point multiplications to compute $D_{i}=w\cdot P$, $E_{i}=w\cdot X$ and $R_{i}=r_{i}\cdot P$.
- Seven hash operations for message authentication and session key generation, including the computation of $a_{i}^{*}$, $HPW_{i}^{*}$, $C_{i}^{*}$, $M_{1}$, $M_{UG}$, $M_{GU}^{*}$, and $SK_{ij}$.

Hence, the total user-side computational cost is: $3T_{M}+7T_{h}$;

The gateway node is responsible for identity recovery, authentication verification, and message forwarding. During the authentication phase, it performs:

- One elliptic curve point multiplication to compute $E_{i}^{*}=x\cdot D_{i}$.
- Eight hash operations to verify message integrity and authenticity, including $K_{GU}$, $M_{UG}^{*}$, $K_{GS}$, $M_{2}$, $M_{GS}$, $M_{SG}^{*}$, $M_{4}$, and $M_{GU}$.

Thus, the computational cost at the gateway node is: $T_{M}+8T_{h}$.

Given the limited computational capability of sensor nodes, the proposed scheme is designed to minimize their cryptographic burden. During the authentication phase, the sensor node performs:

- Two elliptic curve point multiplications to compute $R_{j}=r_{j}\cdot P$ and the shared secret component $r_{j}\cdot R_{i}$.
- Three hash operations for message authentication and session key derivation.

Hence, the computational cost at the sensor node is: $2T_{M}+3T_{h}$.

The computational performance evaluation in this work follows the standard analytical cost estimation methodology widely adopted in lightweight AKA studies. Specifically, the execution times of basic cryptographic primitives are taken from the benchmark results reported in Srinivas et al. [26], obtained on a platform equipped with a 2.4 GHz CPU and 4 GB RAM: the time required for symmetric encryption/decryption $T_{s}\approx 8.7$ ms, the execution time of a one-way hash function $T_{h}\approx 0.32$ ms, the time for ECC point multiplication $T_{M}\approx 17.1$ ms, the time for ECC point addition $T_{A}\approx 4.4$ ms, and the execution time of the fuzzy extractor Gen/Rep function $T_{f}$, which is assumed to be approximately equal to $T_{M}$.

In our system model, the gateway node is assumed to be a relatively resource-rich entity (e.g., an edge server or base station), whereas sensor nodes are resource-constrained devices. Although ECC operations are still required at the sensor side, the proposed protocol is designed to keep the sensor-side computational workload at a minimal level compared with existing schemes, thereby ensuring feasibility in practical WSN environments.

User–gateway–sensor interactions are simulated analytically by sequentially following the authentication message flow ($Msg_{1}$–$Msg_{4}$) and counting the dominant operations executed by each participant. The total protocol cost is obtained by multiplying the operation counts by the corresponding benchmark execution times, providing a fair and reproducible comparison with related protocols.

Table 4 summarizes the computational cost comparison for the authentication phase, contrasting our scheme with four representative related protocols. As shown in Table 4, the proposed protocol exhibits the lowest overall computational cost among the compared schemes, achieving 108.4 ms per authentication session. The computational workload distribution of the proposed protocol is balanced: the gateway performs lightweight verification and forwarding, while the sensor node avoids excessive public-key operations. Such a design is particularly suitable for practical WSN deployments, where sensor-side energy consumption and latency are critical. Overall, the comparison indicates that the proposed scheme achieves competitive efficiency while retaining strong security properties.

### 6.2. Communication Performance Analysis

The communication performance is evaluated by comparing the total number of transmitted bits exchanged during the authentication phase. To ensure a fair comparison, the following assumptions are adopted for data length:

- Timestamp: 32 bits.
- Random number: 256 bits.
- Hash output: 256 bits.
- Identity (ID): 128 bits.
- Elliptic curve point: 256 bits.
- PUF challenge: 128 bits.

During the authentication phase, four rounds of message exchanges are involved:

- $Msg_{1}=\left\{PID_{i},D_{i},M_{1},M_{UG},T_{1}\right\}$ from $U_{i}$ to GW.
- $Msg_{2}=\left\{M_{2},M_{GS},Ch_{j},T_{2}\right\}$ from GW to $S_{j}$.
- $Msg_{3}=\left\{M_{3},M_{SG},T_{3}\right\}$ from $S_{j}$ to GW.
- $Msg_{4}=\left\{M_{4},M_{GU},T_{4}\right\}$ from GW to $U_{i}$.

In these messages, $M_{UG}$, $M_{GS}$, $M_{SG}$, and $M_{GU}$ denote hash values, while $T_{1}$, $T_{2}$, $T_{3}$, and $T_{4}$ represent timestamps. In addition, $D_{i}$ corresponds to a point on the elliptic curve, and $Ch_{j}$ denotes the PUF challenge. The values $PID_{i}$, $M_{1}$, $M_{2}$, $M_{3}$, and $M_{4}$ are obtained through XOR operations, whose lengths are determined by the longer operands involved, resulting in bit-lengths of 256 bits, 384 bits, 384 bits, 512 bits, and 384 bits, respectively. Consequently, the total communication cost of the authentication phase amounts to 256∗4+32∗4+256+128+256+384+384+512+384=3456 bits.

The communication cost comparison between the proposed scheme and the related schemes is summarized in Table 5. As summarized in Table 5, the proposed protocol requires four message rounds, which is consistent with the compared schemes and is generally regarded as a reasonable trade-off between security and latency in WSN scenarios. Although Kumar et al. [25] report a slightly lower communication cost (3200 bits), the proposed protocol achieves substantially lower computational cost and supports richer security functionality. Therefore, from a system-level perspective, the proposed protocol offers a favorable performance trade-off.

## 7. Discussion

The proposed AKA protocol presents a flexible security and efficiency trade-off for wireless sensor networks. The security of the cryptographic techniques is based on the well-established mathematics of elliptic curves. In particular, the sensor node only requires a few elliptic curve operations and hash computations during the authentication phase, which effectively reduces energy consumption.

The storage of long-term secrets in memory is avoided and physical capture and key extraction attacks are prevented to enhance the security of the sensor nodes using Physical Unclonable Functions. Furthermore, in the context of WSNs, dynamic pseudonyms and session-dependent randomness achieve user anonymity and untraceability.

The proposed protocol enhances its features with secure password update function and forgotten password reset. This is different from existing related ones. At the same time, it keeps competitive computational and communication efficiency. The design strikes a good balance among security, usability, and efficiency.

In addition to the analytical performance evaluation, it is important to consider parameter configuration in real deployment scenarios. The gateway node may adopt the widely used elliptic curve secp256r1 to balance security and computational efficiency. Since sensor nodes only perform two ECC scalar multiplications per authentication session, the proposed protocol remains feasible for resource-constrained environments. Furthermore, the timestamp tolerance window ΔT can be set to 2–5 s depending on network latency conditions. A 128-bit PUF challenge length is recommended to enhance resistance against modeling attacks while maintaining low storage overhead. These deployment-oriented considerations further support the applicability of the proposed scheme in practical WSN-based IoT systems.

The proposed protocol is built on ECC, whose security relies on the elliptic curve discrete logarithm problem. It is known that large-scale quantum computers running Shor’s algorithm may threaten ECC-based schemes. Therefore, the current design mainly targets classical security in resource-constrained WSN environments. Nevertheless, the protocol framework is modular, and ECC-based key establishment can be replaced by post-quantum primitives (e.g., lattice-based approaches) in future extensions. This will be considered as an important direction for long-term IoT security.

## 8. Conclusions

In this study, we proposed a secure and efficient AKA protocol for WSNs. The proposed protocol guarantees secure mutual authentication as well as session key establishment. It also offers strong privacy protection. This was achieved through ECC-based smart card user authentication and PUF-assisted sensor authentication.

The proposed protocol was shown to be secure against impersonation, replay, offline password guessing, and stolen smart card attack through formal verification using BAN logic and ProVerif along with informal security analysis. Performance results show that our protocol reduces computation compared with several prior schemes, while keeping communication overhead at an acceptable level.

Therefore, the protocol is well suited to practical WSN scenarios requiring secure, privacy-preserving, and lightweight authentication. Future work on the scheme will include more complex architectures of the network and further improvement in robustness on deployment.

Future work will extend the proposed scheme in two directions: (i) conducting simulation-based WSN evaluations to measure authentication delay and energy consumption under varying node densities and network scales, and (ii) integrating lightweight post-quantum key agreement primitives to improve long-term security against quantum adversaries.

## Figures and Tables

**Table 1:** Basic notations of BAN logic.

Notation	Meaning
P∣≡X	Principal *P* considers *X* to be true
P◃X	Principal *P* has received message *X*
P∣∼X	At some earlier time, Principal *P* uttered message *X*
P⇒X	Principal *P* has jurisdiction over statement *X*
#(X)	Message *X* is fresh (i.e., has not been sent before)
$P\overset{K}{↔}Q$	Principals *P* and *Q* share a valid secret key *K*
${\left\{X\right\}}_{K}$	Message *X* is encrypted using key *K*
(X,Y)	The concatenation of messages *X* and *Y*

**Table 2:** Common inference rules of BAN logic.

Rule	Formal Expression	Explanation
Message Meaning Rule (MMR)	$\frac{P|\equiv P\overset{K}{↔}Q,\;P◃{\left\{X\right\}}_{K}}{P|\equiv Q|∼X}$	If *P* trusts *K* as a shared secret with *Q* and observes ${\left\{X\right\}}_{K}$, then *P* concludes that *Q* previously sent *X*.
Nonce Verification Rule (NVR)	$\frac{P|\equiv \#(X),\;P|\equiv Q|∼X}{P|\equiv Q|\equiv X}$	When *P* regards *X* as fresh and also believes *Q* once stated *X*, *P* can infer that *Q* now believes *X*.
Jurisdiction Rule (JR)	$\frac{P|\equiv Q\Rightarrow X,\;P|\equiv Q|\equiv X}{P|\equiv X}$	If *P* accepts *Q* as an authority on *X* and believes that *Q* believes *X*, then *P* adopts *X* as well.
Freshness Rule (FR)	$\frac{P|\equiv \#(X)}{P|\equiv \#(X,Y)}$	Freshness of *X* implies freshness of the composite (X,Y).
Belief Rule (BR)	$\frac{P|\equiv Q|\equiv (X,Y)}{P|\equiv Q|\equiv X}$	If *P* thinks *Q* believes (X,Y), then *P* can safely infer that *Q* believes *X*.

**Table 3:** The reasoning process based on BAN logic.

**Goals**							
$G_{1}$	$U_{i}|\equiv \left(U_{i}\overset{SK}{↔}S_{j}\right)$			$G_{2}$	$U_{i}|\equiv S_{j}|\equiv \left(U_{i}\overset{SK}{↔}S_{j}\right)$		
$G_{3}$	$S_{j}|\equiv \left(U_{i}\overset{SK}{↔}S_{j}\right)$			$G_{4}$	$S_{j}|\equiv U_{i}|\equiv \left(U_{i}\overset{SK}{↔}S_{j}\right)$		
**Idealizations**							
$Msg_{1}$		$U_{i}\to GW:\;{\left\{\left(R_{i},SID_{j}\right),T_{1}\right\}}_{KGU}$					
$Msg_{2}$		$GW\to S_{j}:\;{\left\{\left(U_{i}\overset{SK}{↔}S_{j}\right),\;\left(U_{i}|\equiv \left(U_{i}\overset{SK}{↔}S_{j}\right)\right),\;T_{2}\right\}}_{KGS}$					
$Msg_{3}$		$S_{j}\to GW:\;{\left\{\left(R_{j},ID_{i},SID_{j}\right),T_{3}\right\}}_{KGS}$					
$Msg_{4}$		$GW\to U_{i}:\;{\left\{\left(U_{i}\overset{SK}{↔}S_{j}\right),\;\left(S_{j}|\equiv \left(U_{i}\overset{SK}{↔}S_{j}\right)\right),\;T_{4}\right\}}_{KGU}$					
**Assumptions**							
$A_{1}$	$U_{i}|\equiv \left(U_{i}\overset{KGU}{↔}GW\right)$			$A_{2}$	$GW|\equiv (U_{i}\overset{KGU}{↔}GW)$		
$A_{3}$	$S_{j}|\equiv \left(S_{j}\overset{KGS}{↔}GW\right)$			$A_{4}$	$GW|\equiv (S_{j}\overset{KGS}{↔}GW)$		
$A_{5}$	$U_{i}|\equiv \#\left(T_{4}\right)$			$A_{6}$	$S_{j}|\equiv \#\left(T_{2}\right)$		
$A_{7}$	$U_{i}|\equiv (GW\Rightarrow (U_{i}\overset{SK}{↔}S_{j}))$			$A_{8}$	$S_{j}|\equiv (GW\Rightarrow (U_{i}\overset{SK}{↔}S_{j}))$		
$A_{9}$	$U_{i}|\equiv (GW\Rightarrow (S_{j}|\equiv \left(U_{i}\overset{SK}{↔}S_{j}\right)))$			$A_{10}$	$S_{j}|\equiv (GW\Rightarrow (U_{i}|\equiv \left(U_{i}\overset{SK}{↔}S_{j}\right)))$		
**Derivation**							
$D_{1}$	From $Msg_{4}$ and $A_{1}$, by MMR:			$U_{i}|\equiv GW|∼\left(T_{4},\;\left(U_{i}\overset{SK}{↔}S_{j}\right),\;\left(S_{j}|\equiv \left(U_{i}\overset{SK}{↔}S_{j}\right)\right)\right)$			
$D_{2}$	From $A_{5}$, by FR:			$U_{i}|\equiv \#\left(T_{4},\;\left(U_{i}\overset{SK}{↔}S_{j}\right),\;\left(S_{j}|\equiv \left(U_{i}\overset{SK}{↔}S_{j}\right)\right)\right)$			
$D_{3}$	From $D_{1}$ and $D_{2}$, by NVR:			$U_{i}|\equiv GW|\equiv \left(\left(U_{i}\overset{SK}{↔}S_{j}\right),\;\left(S_{j}|\equiv \left(U_{i}\overset{SK}{↔}S_{j}\right)\right)\right)$			
$D_{4}$	From $D_{3}$, by BR:			$U_{i}|\equiv GW|\equiv \left(U_{i}\overset{SK}{↔}S_{j}\right)$			
$D_{5}$	From $D_{4}$ and $A_{7}$, by JR:			$U_{i}|\equiv \left(U_{i}\overset{SK}{↔}S_{j}\right)$ (Goal $G_{1}$)			
$D_{6}$	From $D_{3}$, by BR:			$U_{i}|\equiv GW|\equiv \left(S_{j}|\equiv \left(U_{i}\overset{SK}{↔}S_{j}\right)\right)$			
$D_{7}$	From $D_{6}$ and $A_{9}$, by JR:			$U_{i}|\equiv S_{j}|\equiv \left(U_{i}\overset{SK}{↔}S_{j}\right)$ (Goal $G_{2}$)			
$D_{8}$	From $Msg_{2}$ and $A_{3}$, by MMR:			$S_{j}|\equiv GW|∼\left(T_{2},\;\left(U_{i}\overset{SK}{↔}S_{j}\right),\;\left(U_{i}|\equiv \left(U_{i}\overset{SK}{↔}S_{j}\right)\right)\right)$			
$D_{9}$	From $A_{6}$, by FR:			$S_{j}|\equiv \#\left(T_{2},\;\left(U_{i}\overset{SK}{↔}S_{j}\right),\;\left(U_{i}|\equiv \left(U_{i}\overset{SK}{↔}S_{j}\right)\right)\right)$			
$D_{10}$	From $D_{8}$ and $D_{9}$, by NVR:			$S_{j}|\equiv GW|\equiv \left(\left(U_{i}\overset{SK}{↔}S_{j}\right),\;\left(U_{i}|\equiv \left(U_{i}\overset{SK}{↔}S_{j}\right)\right)\right)$			
$D_{11}$	From $D_{10}$, by BR, and $A_{8}$ JR:			$S_{j}|\equiv \left(U_{i}\overset{SK}{↔}S_{j}\right)$ (Goal $G_{3}$)			
$D_{12}$	From $D_{10}$, by BR and $A_{10}$, by JR:			$S_{j}|\equiv U_{i}|\equiv \left(U_{i}\overset{SK}{↔}S_{j}\right)$ (Goal $G_{4}$)			

**Table 4:** Computational cost comparison in the authentication phase with existing schemes [3,23,24,25].

Protocol	User $U_{i}$	Gateway Node GW	Sensor Node $S_{j}$	Total Cost (in ms)
Zhao et al. [23]	$2T_{M}+8T_{h}+T_{f}$	$2T_{M}+8T_{h}$	$2T_{M}+5T_{h}$	126.4 ms
Vangala et al. [24]	$5T_{M}+12T_{h}+T_{A}+T_{s}+T_{f}$	$6T_{M}+12T_{h}+2T_{A}$	$4T_{M}+9T_{h}+T_{A}$	310.5 ms
Huang et al. [3]	$4T_{M}+17T_{h}+T_{f}$	$2T_{M}+17T_{h}$	$3T_{M}+8T_{h}$	180.4 ms
Kumar et al. [25]	$2T_{M}+4T_{h}$	$4T_{M}+9T_{h}$	$2T_{M}+6T_{h}$	142.9 ms
Proposed	$3T_{M}+7T_{h}$	$T_{M}+8T_{h}$	$2T_{M}+3T_{h}$	108.4 ms

**Table 5:** Communication cost comparison in the authentication phase with existing schemes [3,23,24,25].

Protocol	Number of Messages	Total Communication Cost (bits)
Zhao et al. [23]	4	3456 bits
Vangala et al. [24]	4	5152 bits
Huang et al. [3]	4	5504 bits
Kumar et al. [25]	4	3200 bits
Proposed	4	3456 bits

## Data Availability

Data are contained within the article.
